# Some Scientific Aspects of Insanity

**Published:** 1891-10-31

**Authors:** 


### Some Scientific Aspects of Insanity.

II.—Brain and Spinal Cord.

The functions of the brain and spinal cord differ from each other more in degree than in kind. As has been already stated, the gray matter on the surface of the brain is the great storehouse of nervous energy and the great liberator of that energy. Now, for the performance of these two functions, the reception of sensations and the liberation of energy, two areas of the brain, are, roughly speaking, set apart. The greater portion of the brain, which is situated behind the great fissure of Silvius, is the portion of the organ set apart for the reception and perception of sensory impressions. It is to this part of the brain that the great bulk of the ingoing nerve fibres proceed, and in the brain cells of that region most of them terminate. Thus the centres of sensation for the five ordinary senses have their localities in this region. The centre for sight is in the occipital lobe; the centre for smell is on the inner and under surface of the hemisphere in a convolution called the uncinate convolution; the centre for touch is in the same region; the centre for hearing is in the upper convolution of the temporal lobe; and the centre for taste in the lower convolution of the same lobe. If any of these portions of gray matter should chance to become diseased or destroyed, the special sense over which it presides would be wholly or partially lost to the individual.

The outgoing fibres, on the other hand, spring from a well-defined part of the brain in front of the fissure of Silvius. The convolutions of this region have now been so minutely studied, that the various centres of motion from which fibres go to the various part of the body are as distinctly marked off as the counties in an ordinary map of England or Scotland. There is a centre for each group of movements; a centre for movements of the tongue and mouth employed in speech, for the movement of the hand, another for that of the leg, another for the muscles of the trunk, and others for movements of the head and eyes.

I have already said that the central gray matter extends from the middle of the base of the brain to the extremity of the spinal cord. Now, all the fibres from the superficial gray matter of the brain which go outwards end first of all in one or other part of that gray matter; for example, the fibres from the brain which are concerned in the movement of the muscles of one side of the face end in the upper portion of the central gray matter from which again the nerve to the muscles of the face springs. The fibres which direct the movement of the right arm spring from the left side of the brain; in the medulla they cross over to the right side of the spinal cord, and end in the gray matter of the same side of the cord, at a spot, roughly speaking between the shoulders. From that spot arises the nerves of motion for the arm and hand. The fibres for the movement of the right leg would in the same way spring from the left side of the brain, in the medulla cross over to the right side of the cord, and end in the gray matter of the same side of the cord, at a spot situated about what is known as the small of the back. The same rule applies to the ingoing fibres. When a part of the skin on say the left leg is pinched or injured, the sensation is conveyed through the nerve of sensation to a spot in the spinal cord situated about the small of the back. The fibres of the nerve almost immediately cross over to the right side of the spinal cord, and pass up the same side to the right half of the brain. In the same way, when the skin of any other part of the body is touched or pinched, the sensory nerve conveying the impression carries it into the matter of the central gray tube, where it crosses to the opposite side, and is received by the opposite side of the brain. These facts have been experimentally proved over and over again by physiologists, who, by cutting nerves of sensation have rendered the part of the body from which they spring totally destitute of feeling, and who, by cutting the nerve of motion to a limb have paralysed its power of motion. They have also been proved by the results of disease, where the disease has destroyed either the gray matter or the tracts of white matter. The power of originating and conveying motion and of receiving sensations has been lost, and when the tracts of white matter have been invaded by the disease, the power of transmitting motion or sensation has been lost. There is another function which the nervous system exercises, and which is as essential in its way as motion or sensation. It is the power of regulating the nutrition of all parts of the body. This function it performs in two ways intimately associated with one another. The first way is by regulating the amount of blood which flows through the arteries of any limb of the body. This is called the vaso-motor function of the nervous system; and in order that it may be properly performed, there is a special set of nerves, named vaso-motor nerves, which end in the walls of the arteries. The arteries, it will be remembered, are best compared to indiarubber tubes, proceeding from the heart to all parts of the body, branching as they proceed, and consequently diminishing in size the further they proceed from the heart. The calibre of these tubes is very materially affected in size by the action of the nervous system. It is well illustrated in the operation of blushing, where an emotion, acting on the nervous system, causes a dilation of the blood vessels of the face and neck. The opposite condition is well illustrated in the action of the emotion of fear, and sometimes of anger, in contracting these arteries, and thus causing pallor of the face. When it is remembered that the blood is the medium through which nourishment is carried to every part of the body, it will be easily understood how this vaso-motor function of the nervous system can affect by either diminishing or increasing the supply of blood to a limb or an organ the nutrition of that limb or organ. But there is another way, and a more mysterious way, in which the nervous system presides over the nutrition of the body. It is called its trophic function. When the anterior or motor root of a spinal nerve is cut, the muscles of the limb to which it supplies motor energy become flabby, and degenerate rapidly. Now, this in no way depends upon any vaso-motor influence, but simply upon a cessation of the outgoing nervous energy or tone. In the same way the activity of the various glands and organs of the body, such as the liver, the stomach, the salivary glands, and others that might be mentioned, are affected apart from vaso-motor changes by direct stimulation through special nerves.

We have now seen the various ways in which the nervous system communicates with the outside world, or rather, by which messages from the outside world are received by the nervous system, and the manner in which the nervous system employs its appendages, namely, the limbs, with their muscles and bones, in acting upon the outside world by means of locomotion and prehension. But there is another system of messages conveyed to the nervous system, namely, those ingoing from every part and organ of the body. There are, therefore, two circuits of nervous energy, the first having its origin in the surroundings in the outside world passing through the ingoing nerves to the central nervous system, and there causing changes (sensations, perceptions, ideas, judgments), which enable the individual by means of the outgoing nerves (nerves of motion) to adapt himself to the constantly changing sequence of events in his surroundings. The second nervous circuit has its origin in the various limbs, bones, joints, integuments, and organs of the body, conveying through ingoing nerves impressions to the central nervous system of the order or disorder of these various parts, and causing changes to take place in the gray matter, by means of which outgoing currents through trophic or vaso-motor nerves, modify, control, or arrest changes in these various organs and parts. Through an infinity of ages and generations these workings of the nervous system have become so admirably proportioned and balanced that in a sane and otherwise healthy individual no single department or function is prominently felt at one given time, but the whole is blended into an exquisitely harmonious feeling, which we call consciousness.

